# Ten Years of the Synthetic Biology Summer Course at Cold Spring Harbor Laboratory

**DOI:** 10.1021/acssynbio.4c00276

**Published:** 2024-09-20

**Authors:** Karmella A. Haynes, Lauren B. Andrews, Chase L. Beisel, James Chappell, Christian E. Cuba Samaniego, John E. Dueber, Mary J. Dunlop, Elisa Franco, Julius B. Lucks, Vincent Noireaux, David F. Savage, Pamela A. Silver, Michael Smanski, Eric Young

**Affiliations:** 1 Wallace H. Coulter Department of Biomedical Engineering, Emory University, Atlanta, Georgia 30345, United States; 2 Department of Chemical Engineering, University of Massachusetts Amherst, Amherst, Massachusetts 01003, United States; 3 Helmholtz Institute for RNA-Based Infection Research (HIRI), Helmholtz Centre for Infection Research (HZI), 97080 Würzburg, Germany; 4 Medical Faculty, University of Würzburg, 97080 Würzburg, Germany; 5 Biosciences Department, Rice University, Houston, Texas 77005, United States; 6 Department of Computational Biology, Carnegie Mellon University, Pittsburgh, Pennsylvania 15213, United States; 7 Department of Bioengineering, University of California Berkeley, Berkeley, California 94720, United States; 8 Biomedical Engineering, Boston University, Boston, Massachusetts 02215, United States; 9 Mechanical and Aerospace Engineering, Bioengineering, University of California, Los Angeles, California 90095, United States; 10 Department of Chemical and Biological Engineering and Center for Synthetic Biology, Northwestern University, Evanston, Illinois 60208, United States; 11 School of Physics and Astronomy, University of Minnesota, Minneapolis, Minnesota 55455, United States; 12 Department of Molecular and Cell Biology, University of California Berkeley, Berkeley, California 94720, United States; 13 Howard Hughes Medical Institute, University of California Berkeley, Berkeley, California 94720, United States; 14 Department of Systems Biology, Harvard Medical School, Boston, Massachusetts 02115, United States; 15 Department of Biochemistry, Molecular Biology, and Biophysics and Biotechnology Institute, University of Minnesota, Minneapolis, Minnesota 55455, United States; 16 Chemical Engineering, Worcester Polytechnic Institute, Worcester, Massachusetts 01609, United States

**Keywords:** education, TXTL, DNA construction, gene regulation, computational modeling, CRISPR

## Abstract

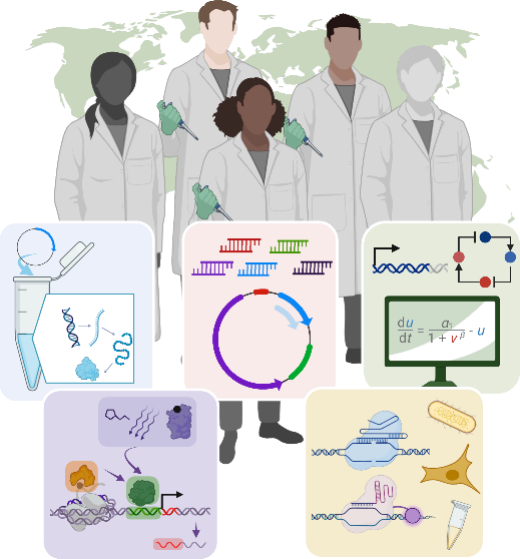

The Cold Spring Harbor Laboratory (CSHL) Summer Course on Synthetic Biology, established in 2013, has emerged as a premier platform for immersive education and research in this dynamic field. Rooted in CSHL’s rich legacy of biological discovery, the course offers a comprehensive exploration of synthetic biology’s fundamentals and applications. Led by a consortium of faculty from diverse institutions, the course structure seamlessly integrates practical laboratory sessions, exploratory research rotations, and enriching seminars by leaders in the field. Over the years, the curriculum has evolved to cover essential topics such as cell-free transcription–translation, DNA construction, computational modeling of gene circuits, engineered gene regulation, and CRISPR technologies. In this review, we describe the history, development, and structure of the course, and discuss how elements of the course might inform the development of other short courses in synthetic biology. We also demonstrate the course’s impact beyond the lab with a summary of alumni contributions to research, education, and entrepreneurship. Through these efforts, the CSHL Summer Course on Synthetic Biology remains at the forefront of shaping the next generation of synthetic biologists.

## 1 Introduction

Cold Spring Harbor Laboratory (CSHL) is a world-renowned private, nonprofit research facility steeped in a rich history of over a century of fundamental biological discoveries. Key milestones in molecular biology were achieved in laboratories within picturesque cottage-style buildings around the harbor. Since Nobel laureate Max Delbrück’s Phage Course in 1945,^1^ CSHL has broadened its impact to scientists and students through its summer courses. Today CSHL offers 30 advanced courses led by visiting professors from other institutions, covering cancer, cell biology, molecular bioinformatics, neurobiology, genetics, and immunology. In 2013, synthetic biology was added to CSHL’s collection of distinguished summer courses, and has formed a collaborative relationship with the highly recognized Yeast Genetics and Genomics course.^2^

The Cold Spring Harbor Summer course in synthetic biology originated from discussions at the 2011 Cold Spring Harbor Asia (CSHA) Symposium on Design & Synthesis of Biological Systems in Suzhou, China, that included conference Co-Chair Pamela Silver, invited speakers Jeff Tabor, Julius Lucks, and David Savage, and CSHA President David Stewart. The idea of a summer course for synthetic biology was pitched during a social gathering on the last day of the symposium by D. J. Stewart, who recruited the founding instructors including J. Tabor, J. Lucks, and D. F. Savage. After returning to the US, the team recruited Karmella Haynes, gathered at a site visit at CSHL, and launched the first course in 2013. Haynes, Lucks, Savage, and Tabor formalized the instruction of synthetic biology into a two-week hands-on course held at a CSHL teaching lab. Since then, the annual course has offered students the opportunity to learn techniques and perform research at the forefront of synthetic biology. Over the past ten years, the course has evolved into an immersive lab experience that teaches how biological system complexity combined with engineering approaches leads to new design principles for bioengineering.

The year 2023 marked the ten-year anniversary of the CSHL Summer Course on Synthetic Biology, which has taken place every year except 2020 and 2021 (during the COVID pandemic). This article highlights historical milestones of the development of the course structure and curricula. We aim to provide readers, especially those who have not yet attended, a view into the course experience. We also aim to provide a useful framework for instructors who are developing synthetic biology short-courses at their own institutions.

## 2 Course Leadership: Instructors, Staff, and Teaching Assistants

In 2013 four faculty, K. Haynes, J. B. Lucks, D. F. Savage, and J. Tabor, from four different institutions, laid the groundwork for the course through online meetings. The course continues to be team-directed by three to five instructors each year. Instructors are responsible for evaluating applications and admitting students, inviting and hosting seminar speakers, and scheduling classes and special events. CSHL staff including Barbara Zane (Manager, Course Planning and Scientific Operations) and a dedicated student intern course assistant work with the instructors to manage lab operations. Prospective new instructors are often invited as seminar speakers to experience the course first-hand and be inspired to return as instructors. As a result of our recruitment efforts, our instructor pool now includes 21 faculty from 15 different institutions.

The Teaching Assistants (TAs), usually graduate students or postdoctoral researchers recruited from the Instructors’ laboratories, serve a critical role in constructing and facilitating the courses. TAs have been instrumental in developing protocols and technical lectures in the months leading up to each course. At CSHL they set up and manage experiments, and provide most of the direct instruction of students. Our pioneering 2013 TAs included four PhD students: Rene Davis, Dana Nadler, Evan Olson, and Melissa Takahashi.

## 3 Course Structure: Development and Broader Utility

### 3.1 Structure of the Two-Week Course

Capturing the diversity and evolving nature of synthetic biology within a two-week hands-on course was a formidable challenge initially. Unlike traditional CSHL courses like yeast genetics and neuroscience, which have well-established techniques and concepts, synthetic biology is a nascent, rapidly evolving field without such foundations. To address this gap, the founding instructors devised a strategy to teach lab fundamentals in parallel microsessions called Practicals, followed by Research Rotations where students apply what they’ve learned to explore new applications or research questions. Inspired by a “learn-by-doing” approach from the Woods Hole Cell Physiology course,^3^ instructors bring active research projects from their laboratories so that Research Rotations can evolve as the field evolves.

As the course developed over subsequent years, a learning experience akin to the progression from college to graduate school took shape. During week one Practicals, i.e., “college”, 16 students are divided into three class sections (Table 1). Each day a section attends one of four different lecture/lab classes on essential synthetic biology techniques, and three classes are run in parallel. During week two Research Rotations, i.e., “graduate school”, each instructor and teaching assistant facilitates a multiday project based on research from their home laboratories (Research Rotation 1–4). The students are asked to choose a research topic offering that aligns with their own interests, and they are reorganized into new groups (Groups A1–D1) that work together for 3 days on a project. For the next rotation, students choose a second topic, are reorganized into new groups (Groups A2–D2), and work together for the next 3 days on a new project.

**Table 1:** Structure of the Two-Week Cold Spring Harbor Summer Course in Synthetic Biology^a^

Sunday	Monday	Tuesday	Wednesday	Thursday	Friday	Saturday
**Week 1: Practicals**						
		Course introduction and reception	P1 - (none)	P1 - Section C	P1 - Section B	P1 - Section A
			P2 - Section A	P2 - (none)	P2 - Section C	P2 - Section B
			P3 - Section B	P3 - Section A	P3 - (none)	P3 - Section C
			P4 - Section C	P4 - Section B	P4 - Section A	P4 - (none)
			Seminar	Seminar	Seminar	Seminar
**Week 2: Research Rotations**						
Free time	Research Rotation 1 - Group A1			Research Rotation 1 - Group A2		
	Research Rotation 2 - Group B1			Research Rotation 2 - Group B2		
	Research Rotation 3 - Group C1			Research Rotation 3 - Group C2		
	Research Rotation 3 - Group D1			Research Rotation 3 - Group D2		
	Seminar (each evening)			Seminar (each evening)		
**Week 3: Course Conclusion**						
Extra day for lab work	Presentations: Groups A1–D1, A2–D2					
	Graduation ceremony					

a P1, 2, 3, or 4 = a Practical focused on one of four topics described under Curriculum Content.

To enhance the learning experience, each evening an internationally recognized invited speaker from academia, industry, or science policy sectors presents a seminar to demonstrate the real-world impact of the course material. At the conclusion of the course, we celebrate the students’ successes with a project showcase (presentations), and a private graduation ceremony where each student receives a certificate signed by the instructors.

### 3.2 Adapting the Course Structure for Other Educational Contexts

We have developed a dual-phase structure that can potentially be adopted for other teaching and training contexts. The first week’s structured Practicals teach essential techniques through parallel microsessions, ensuring all students gain core skills. The Practicals phase is designed to cover four independent, yet complementary topics (discussed in the next section), allowing instructors to change or update specific content to keep pace with the rapidly evolving field of synthetic biology. The second week’s Research Rotations allow students to apply these skills to exploratory group projects, reinforcing comprehension of the core skills from week one. The short duration and efficiency of this dual-phase structure gives it the potential to be adapted for professional training workshops, or institutional courses led by teams of instructors. For professional training of educators, technicians, and industry scientists, the dual-phase structure could be used without modifications in a dedicated two-week retreat-style setting. Implementation in a professor team-led college or graduate school course that typically meets only twice a week may require an extended timeline of at least 4 weeks. It may be possible to maintain the structure of the Practicals as parallel one-day microsessions, and to implement the Research Rotations as small group projects that apply skills learned from the Practicals. This course structure might not be suitable for K-12 education, which typically relies on reinforcement of concepts over a longer period of time.^4^ In summary, the CSHL Synthetic Biology summer course effectively balances foundational learning with hands-on research, providing a model that could be suitable for advanced (post K-12) synthetic biology courses.

## 4 Curriculum Content

**Figure 1:**
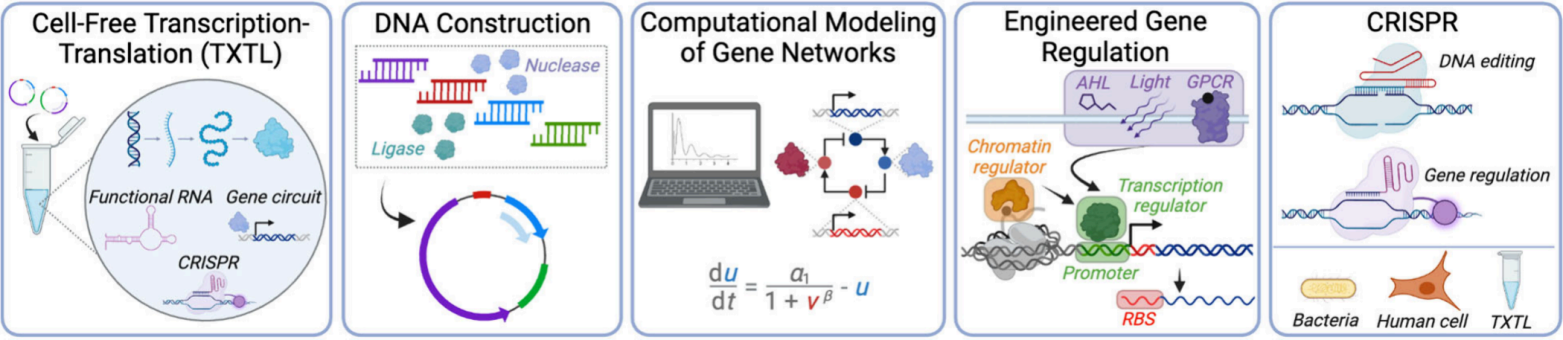
Major themes addressed by the CSHL Summer Course curriculum from 2013–2023. Figure created with BioRender.com.

In the first year of the course, the curriculum included four primary themes: cell-free transcription and translation, DNA construction, computational modeling of gene networks, and engineered gene regulation in bacteria-based systems. In subsequent years, additional themes emerged, such as CRISPR-based bioengineering tools, as well as gene regulation and editing for specific host systems including yeast and mammalian cells (Figure 1).

### 4.1 Cell-Free Transcription–Translation (TXTL)

Experimentally characterizing the outputs of genetic parts and networks is a critical stage of bioengineering. Such experiments provide quantitative data to guide mathematical models, assess the performance of genetic circuits, and enable iteration through the design-build-test-learn (DBTL) cycle. To create an efficient prototyping platform, synthetic biologists have harnessed cell-free transcription–translation (TXTL) systems.^5,6^ TXTL achieves *in vitro* gene expression without the need to deliver DNA into host cells. Furthermore, some applications use PCR-amplified or *in vitro*-assembled DNA to bypass upstream cloning steps, such as the PHage Engineering by *In Vitro* Gene Expression and Selection (PHEIGES) system.^7^ Thus, TXTL enables rapid DBTL cycles that test artificially constructed networks of interacting compounds, proteins, and nucleic acids in hours instead of days.^8,9^

Inspiration to develop a TXTL course topic came from interactions between J. B. Lucks, Vincent Noireaux, and Richard Murray at the February 2013 DARPA Living Foundries meeting, where they discussed leveraging the experimental accessibility and rapid turnaround time of TXTL systems as an engine for teaching. J.B. Lucks used TXTL to demonstrate the performance of RNA-based transcriptional repressors and signaling networks in 2013 and used TXTL for cell-free biosensing in 2015. In 2017, V. Noireaux and Chase Beisel collaborated to integrate CRISPR into TXTL to demonstrate rapid cell-free characterization of gRNAs and Cas proteins.^10,11^ Genetic circuits based on small transcription-activating RNA (STARs) were incorporated into the curriculum by James Chappell in 2018.^12^ Then in 2022, V. Noireaux used cell-free synthesis of infectious bacteriophages to show that large DNAs can also be employed in TXTL.^13^

TXTL has been a powerful platform for synthetic biology education, especially within a short time frame.^14^ The speed at which TXTL experiments can yield results (i.e., hours) and its user-friendly setup make it ideal as an educational tool, allowing course participants flexibility in their experimental design and the ability to iterate through many experimental questions in a matter of days.

### 4.2 DNA Construction

At the core of synthetic biology is the genetic coding of biological devices to perform a desired function. Producing biological devices with predictable behavior is a persisting challenge in synthetic biology. Accordingly, efficient DBTL cycles usually require an empirical process where many constructs must be built with variations in specific parameters. Due to this near-universal component of a synthetic biology project, a DNA construction topic has been featured every year since 2013.

DNA construction was introduced in 2013 by D. F. Savage who used Golden Gate to teach students single step digestion-ligation assembly of plasmids for bacterial expression of pigment synthesis genes including violacein and indigo. Later, John Deuber related the concepts of assembling characterized parts into composite parts and genetic devices using a MoClo Golden Gate cloning strategy with a toolkit of characterized yeast parts.^15^ In subsequent years, Michael Smanski and Lauren Andrews introduced hierarchical DNA assembly strategies to create large multipart constructs, such as genetic circuits,^16,17^ and used acoustic liquid handling automation for library-scale construction. The latest iteration of this curriculum developed by Eric Young now includes Golden Gate assembly of a combinatorial pathway library, where students vary expression elements to find the optimal design through linear regression.

To enhance the learning experience, we ultimately seek to shift the paradigm of DNA design from a process that makes incremental changes to plasmids or backbones that already exist to a process of complete rational design, where every single base pair of a DNA construct is mutable. This allows students to think intentionally about every part in a construct and empowers them to optimize the construct for their own scientific objective. Strengths and weaknesses of different assembly techniques are discussed so that students can choose the most efficient method.^18^ A highly engaging exercise that was developed by M. Smanski for the DNA construction curriculum is the Five Primer Challenge.^19^ Students are given a plasmid that expresses green fluorescent protein (GFP) at moderate levels, up to five 60-mer oligonucleotides of their own design, and 3–4 days to engineer the brightest GFP strain of *E. coli* possible. This exercise has yielded an impressive array of clever DNA assembly strategies, demonstrating how the curriculum fosters creative problem-solving.

### 4.3 Computational Modeling of Gene Circuits

Mathematical modeling plays a critical role in the rational design and efficient optimization of synthetic gene circuits. For example, early synthetic circuit designs were often motivated by theory or simulations based on mathematical analysis to predict bistability or oscillatory behavior.^20^ As the synthetic biology field has progressed, computational modeling efforts have expanded dramatically to include temporal and spatial models, stochastic systems, and machine learning models. The tight integration of modeling and experiments in synthetic biology motivated the need for modeling-based instruction within the CSHL Synthetic Biology course.

In 2015, Mary Dunlop introduced computational modeling for gene circuits as a topic and it has been represented continuously in the course curriculum since that point. In the beginning, topics included an overview of ordinary differential equation models and instruction on methods for developing systems of equations for gene regulation. This included coverage of topics like Michaelis–Menten reaction kinetics. Instructors including Elisa Franco and Christian E. Cuba Samaniego continued to develop hands-on coding lessons, teaching students how to simulate classical synthetic circuit models like the repressilator^21^ using Matlab or Python. Modeling of multicellular systems has also been introduced into the curriculum by Ophelia Venturelli. Recently, C. E. Cuba Samaniego developed an experimental component so that modeling can be integrated into the Research Rotations (week two) for design of synthetic genetic circuits.

Overall, mathematical modeling for synthetic circuit design remains a core area of importance, and the course aims to provide students with a straightforward entry point to understand models that they will encounter in the literature, while inspiring students to expand their skills in this area.

### 4.4 Engineered Gene Regulation and CRISPR Technologies

Curricula focusing on the topic of engineered gene regulation was integral to the initial development of the course. Content under this area has included a diverse array of specific synthetic biology applications, which often integrate core concepts from the previous three areas, i.e., TXTL, DNA construction, and computational modeling of gene circuits. The topic of CRISPR technologies was introduced in year two (2014) in response to the emerging importance of this area in synthetic biology. In subsequent years, engineered gene regulation and CRISPR technologies have been taught interchangeably.

#### 4.4.1 Engineered Gene Regulation

Given the central role of transcriptional states (i.e., active and repressed) of DNA in biology, gene regulation remains a cornerstone subject in synthetic biology. Learning how to achieve precise control of the magnitude and timing of gene expression enables construction and operation of synthetic circuits, or the regulation of host cell genes within chromosomal DNA. Our curriculum has demonstrated how engineered gene regulation enables tailored manipulation of cells, and has expanded host cells used in the course from bacteria to eukaryotes including yeast and human tissue culture.

Our aim has been to revisit the foundational concepts of gene regulation within the context of biotechnology, and to introduce students to recent innovations that harness underutilized mechanisms for customizable systems. In 2013 engineered regulation was explored using chemical and light inducers. K. Haynes used bacterial quorum sensing to explore orthogonal signaling and crosstalk with a panel of acyl homoserine lactone (AHL) producing “sender” enzymes tested against a diverse set of AHL-sensitive GFP-expressing reporters.^22^ J. Tabor used optogenetics to demonstrate how light-sensitive regulators enable precise temporal control of engineered genes in bacteria.^23^ Since then, tunable gene regulation tools including customizable noncoding elements (promoters and ribosome binding sites), CRISPR-activation and inhibition, engineered chromatin proteins, and engineered G-coupled protein receptor-regulators were introduced by Howard Salis, C. L. Beisel, K. Haynes, Ahmad Khalil, and Pamela Peralta-Yahyah. High-throughput microfluidics to evaluate engineered gene expression was introduced by Philip Romero.

Teaching gene regulation in the context of bioengineering supports deeper understanding of underlying mechanisms by tuning the activities of key components, such as promoters, regulator proteins, and signal receptors, and observing how changes in DNA sequences and protein structure affect gene expression dynamics. We hope that this training empowers scientists to develop new bioengineering tools that overcome limitations of many long-established systems, such as tetracycline-inducible promoters.

#### 4.4.2 CRISPR Technologies

CRISPR technologies have revolutionized the field of synthetic biology by providing unprecedented flexibility in customizable sequence targeting and DNA or RNA editing. These technologies typically rely on a CRISPR-associated (Cas) nuclease such as Cas9 and a programmable guide RNA to direct the binding and cutting of a selected sequence.^24^ CRISPR technologies have become a mainstay of our curriculum. CRISPR-focused Practicals and Rotations have evolved to track with the rapid evolution of this area.

To capture these technologies in the CSHL curriculum, CRISPR was introduced by K. Haynes in 2014 for the optimization of targeted genome mutagenesis in cultured human cells. The CRISPR curriculum was expanded in 2016 to include programmable gene repression in *E. coli* and prototyping in TXTL. Combining CRISPR and TXTL had never been reported before, and this novel effort became the basis of the first collaborative publication of its kind from V. Noireaux and C.L. Beisel.^10^ In subsequent years, the course continued to be a testbed for different ways to use CRISPR in a cell-free context, such as testing guide RNA activity, screening novel nucleases, generating CRISPR-based circuits, and testing putative CRISPR inhibitor proteins.

The CRISPR testbeds featured in our curriculum have leveraged *E. coli* and TXTL to provide an accessible platform for students to learn the fundamentals of CRISPR customization. These fundamental lessons can inform other specialized uses in other host cells and species. In addition, students have been introduced to the latest CRISPR advances, such as base editing, prime editing, and CRISPR transposition. By doing so we hope to inspire students to contribute to the ongoing development of this powerful technology by generating their own innovations to improve the precision and portability of CRISPR across different contexts.

### 4.5 Adapting the Curriculum Content for Other Synthetic Biology Courses

Our central educational goal, which is applicable to other synthetic biology courses, is to provide students with a comprehensive and practical understanding of synthetic biology principles and techniques. Therefore, the curriculum serves as a model for other courses, for instance, institutional classes and professional workshops. Our educational goal is achieved through a hands-on, iterative learning approach that includes cell-free transcription and translation (TXTL), DNA construction, computational modeling, and genetic manipulation in host cells (gene regulation and CRISPR), allowing students to rapidly prototype, test, and refine engineered biological systems. Each year, alumni are given a manual of experiments and protocols, which they could use to integrate parts of the curriculum into other courses. To support broader accessibility of this material, it will be important to develop a manual such as the protocol book edited by instructors from the CSHL Advanced Bacterial Genetics summer course, and recently published by Cold Spring Harbor Laboratory Press.^25^

## 5 Invited Speakers for Curricular Enrichment

Each evening, our course features focused seminars delivered by junior and senior academic faculty and industry leaders in synthetic biology and bioengineering, enriching the educational experience with experiential insights and practical applications. These seminars, typically featuring 9–12 speakers per year, delve into topics that complement and expand upon the fundamental curriculum. They cover a wide array of subjects including host cells not covered in the lab modules, industrial applications of synthetic biology, advancements in agriculture, and discussions on the societal impacts and ethical considerations of bioengineering. Our inaugural year (2013) included an exceptional lineup of speakers including Richard Murray, Eric Klavins, Pamela Silver, Harris Wang, Adam Arkin, Dan Gibson, Andy Ellington, Michelle Chang, Justin Gallivan, Mike Jewett, Ron Weiss, and Megan Palmer. Notably, we’ve had the privilege of hosting distinguished guests such as Amyris founder Jay Keasling in 2014 and 2023, Nobel Prize laureate Francis Arnold in 2018, and Chief Scientific Officer of Eden Brew, Caludia Vickers in 2023. In total, invited speakers from 2013 to 2023 included 65 different academic faculty and 6 industry professionals from 47 different universities and 5 different companies.

## 6 Students, Alumni, and Impacts on Career Development

**Figure 2:**
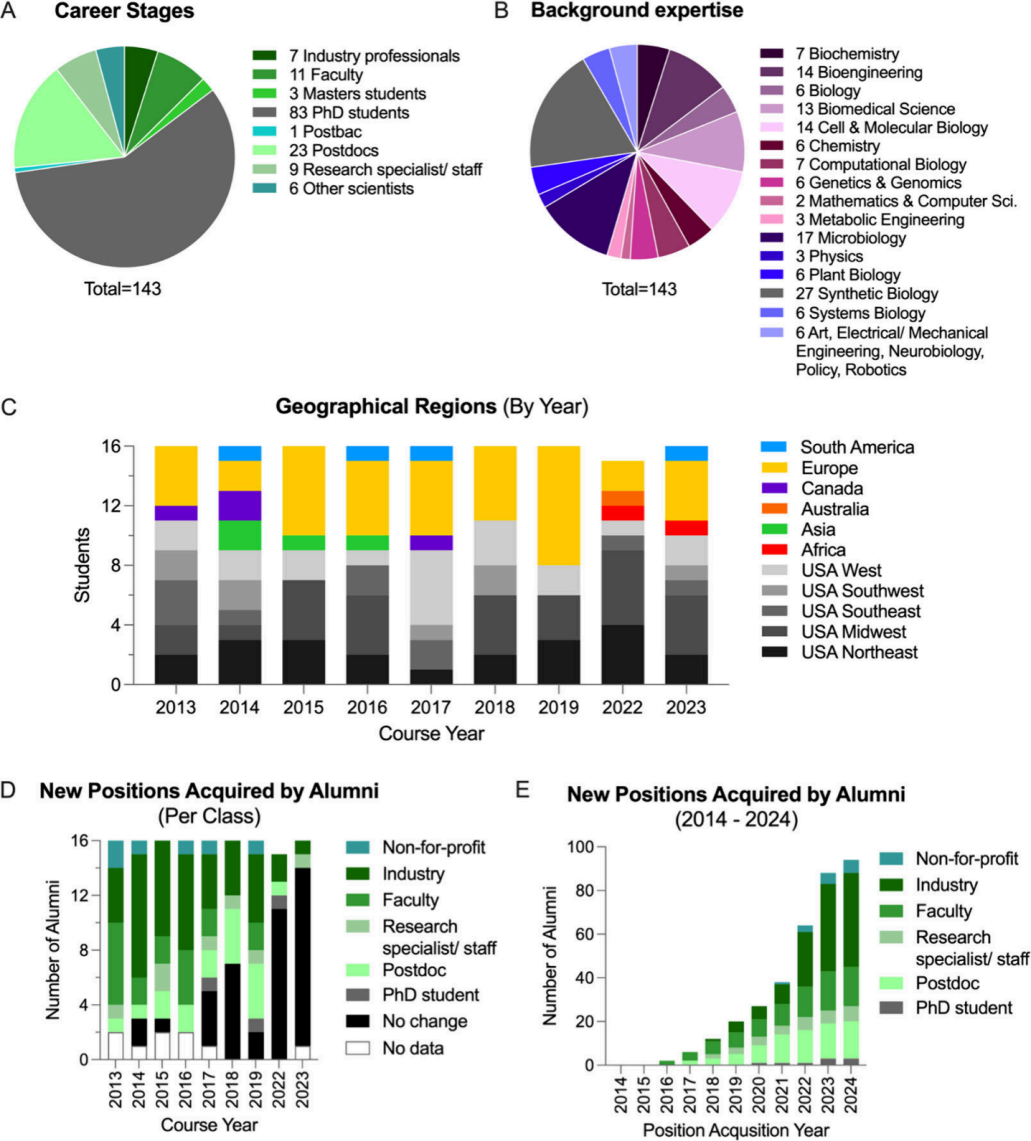
Career stages, backgrounds, geographical representation, and postcourse career advancement of 143 student alumni from 2013–2023. (A) Professional stages of students. (B) Areas of expertise represented by students at the time of course attendance. (C) Geographical representation by year. (D) Numbers of alumni from each class who acquired new positions in synthetic biology-related fields as of 2024. (E) The cumulative totals of new positions acquired by alumni from 2014–2024. Data for D and E were collected from online profiles at LinkedIn and institutional Web sites where available.

Our students and alumni consist of scientists at various stages of their careers, with the majority being PhD students (58% overall, 2013–2023) (Figure 2A). The instructors typically select applicants whose scientific training and careers are well underway, including advanced graduate students, postdoctoral fellows, lab technicians, faculty, and professionals from industry and the not-for-profit sector. By doing so, we aim to support nascent trajectories toward work and careers in synthetic biology. Although most students have technical backgrounds in synthetic biology (19%), bioengineering (10%), and cell and molecular biology (10%), many represent a diverse array of other focus areas including art, biomedical science, chemistry, mathematics and computer science, physics, and more, reflecting the interdisciplinary spirit of the course (Figure 2B). Our students have come from different regions across the globe, with the majority from the U.S. (61%) due to fellowship eligibility requirements (Figure 2C).

The majority of course alumni continued their current positions or acquired new positions in fields related to synthetic biology (Figure 2D). This degree of retention within bioengineering may be due to students’ pre-existing interest in synthetic biology, their interest in enhancing their current work, or their motivation to advance their careers in bioengineering. Alumni who did not change positions already held advanced positions, including faculty from 2014 and a postdoc from 2017, at the time they attended the course. Most who have not yet secured new positions were students from more recent courses. Several alumni acquired new positions as PhD students (3 total), or advanced to postdoctoral fellowships (17 total), suggesting that the CSHL course helps applicants become more competitive for selective training programs. Overall, 43 of 143 alumni acquired research and development, consultant, or sales positions in industrial biotechnology (Figure 2E) at companies including Andreessen Horowitz, Boehringer Ingelheim, Cooley LLP, Corteva Agroscience, Exxon, Ginkgo Bioworks, Lonza, Novartis, Novozymes, Sartorius, and many others. Industry is the most represented area in newly acquired positions, suggesting that the skills and knowledge gained from the course are highly valued by leading companies in the biotech sector.

Several course participants and alumni have acquired leadership positions in synthetic biology. Past TAs including Melissa Takahashi (2013), James Chappel (2015), and C. E. Cuba Samaniego (2017) now hold academic faculty positions, and Shakked Halperin (2018) founded a company, Rewrite Therapeutics. Their current work is related to research and technologies from past CSHL courses. Graduate students, postdocs, and other scientists who matriculated the course from 2013–2023 have started or advanced to leadership positions in different sectors. Sixteen alumni advanced to positions as faculty in academia. Many have secured leadership positions in industry and venture capitalism (20 total), and not-for-profit organizations (4 total) related to bioengineering. Student alumni have also applied their education and new network connections to launch or advance new companies and programs. These include: Stemloop, a biosensor development company; General Biological, a bioproduction company; SynBio Africa, a not-for-profit research initiative; Santa Ana Bio, an immunotherapy development company; and the Frugal Science Academy at Georgia Tech which uses TXTL to develop accessible science education.^14^

Course impacts also include published research that has come directly from work done during the summer course, advancement of careers in synthetic biology, and the creation or advancement of new synthetic biology programs. Gene constructs and data produced during the course have spurred research publications with instructors, TAs and students as coauthors. In 2013, a major insight of the in-course work with TXTL was to uncover, for the first time, the fast dynamics of RNA-only transcriptional networks, which resulted in a publication.^8^ Also that year, the gene regulation quorum sensing system used for the CSHL course supported a project and publication from the international Genetically Engineered Machines Competition (iGEM) team at Arizona State University.^26^ The first introduction of CRISPR systems in 2014 led to a publication that reported the impact of chromatin on DNA editing efficiency in human cells.^27^ A topic on cell-free biosensing in the 2015 course led to a new platform diagnostic technology called ROSALIND by one of the course students.^28^ In the 2017 course, an idea hatched as part of the CRISPR curriculum became the basis of RNA-sensing Cas12a nucleases published two years later.^29^

## 7 Conclusion: Vision of the Course’s Future

The first ten years of the CSHL Synthetic Biology summer course have covered four technical themes, cell-free transcription–translation (TXTL), DNA construction, computational modeling of gene circuits, and engineered gene regulation and CRISPR technologies, to lay a solid foundation for future growth and adaptation to the rapidly evolving field. Looking ahead, the course can further align with cutting-edge advancements by incorporating new themes that reflect other broadly useful, fundamental concepts in synthetic biology. For instance, directed evolution, which was highlighted in the 2016 course by Harris Wang^30^ and by invited speaker Francis Arnold (2018), exemplifies the power of harnessing evolutionary principles for bioengineering. This approach allows for the production of novel proteins and metabolic pathways through an iterative selection process, enabling breakthroughs in medicine, environmental sustainability, and industrial biotechnology. Second, microfluidics and other automated liquid handling techniques represent a transformative shift in experimental methodology for synthetic biology.^31^ Microfluidics allows for precise control of small volumes of fluids, facilitating high-throughput screening and rapid prototyping of synthetic constructs. This technology can significantly accelerate research and development cycles, supporting efficiency in the application of the design-build-test-learn cycle of synthetic biology. Third, exploring the regulation of multicellular communities would expand the scope of the current course material from the molecular level to the cellular level. Designing cell interactions within populations, for instance via engineered mammalian Notch/Delta or microbial quorum sensing pathways,^32,33^ opens new avenues for applications in tissue engineering and bioproduction. Finally, to promote diversity and inclusion, we aim to expand the geographical representation of both students and instructors to foster the global development of synthetic biology education, research, and applications. Through these initiatives, we aspire to equip the next generation of synthetic biologists with the knowledge, skills, and resources necessary to address complex challenges and drive transformative discoveries.
